# Thrombin Generating Capacity and Phenotypic Association in ABO Blood Groups

**DOI:** 10.1371/journal.pone.0141491

**Published:** 2015-10-28

**Authors:** Romy M. W. Kremers, Abdulrahman B. O. Mohamed, Leonie Pelkmans, Salwa Hindawi, H. Coenraad Hemker, H. Bas de Laat, Dana Huskens, Raed Al Dieri

**Affiliations:** 1 Synapse, Cardiovascular Research Institute Maastricht, Maastricht University, Maastricht, the Netherlands; 2 Department of Pharmacology, King Abdulaziz University, Jeddah, Saudi Arabia; 3 Department of Haematology, King Abdulaziz University, Jeddah, Saudi Arabia; 4 Department of Biochemistry, Cardiovascular Research Institute Maastricht, Maastricht University, Maastricht, the Netherlands; National Cerebral and Cardiovascular Center, JAPAN

## Abstract

Individuals with blood group O have a higher bleeding risk than non-O blood groups. This could be explained by the lower levels of FVIII and von Willebrand Factor (VWF) levels in O individuals. We investigated the relationship between blood groups, thrombin generation (TG), prothrombin activation and thrombin inactivation. Plasma levels of VWF, FVIII, antithrombin, fibrinogen, prothrombin and α_2_Macroglobulin (α_2_M) levels were determined. TG was measured in platelet rich (PRP) and platelet poor plasma (PPP) of 217 healthy donors and prothrombin conversion and thrombin inactivation were calculated. VWF and FVIII levels were lower (75% and 78%) and α_2_M levels were higher (125%) in the O group. TG is 10% lower in the O group in PPP and PRP. Less prothrombin was converted in the O group (86%) and the thrombin decay capacity was lower as well. In the O group, α_2_M plays a significantly larger role in the inhibition of thrombin (126%). In conclusion, TG is lower in the O group due to lower prothrombin conversion, and a larger contribution of α_2_M to thrombin inactivation. The former is unrelated to platelet function because it is similar in PRP and PPP, but can be explained by the lower levels of FVIII.

## Introduction

The hemostatic system should keep blood in a fluid state under normal conditions and minimize blood loss via the arrest of bleeding at sites of vascular injury. It is a complex web of reactions in which dozens of components play a role. Uncontrolled activation of the system may result in the occlusion of the vascular system, i.e. thrombosis and embolism. On the other hand, serious haemorrhage threatens if the hemostatic system fails.

A close relationship exists between ABO blood type and the risk of venous thromboembolism [1–3], coronary heart disease [1, 4] and atherosclerosis [5, 6]. An increased bleeding tendency has been reported for blood group O [7, 8] and in patients with inherited bleeding disorders, individuals with blood group O are consistently overrepresented [7–9]. ABO blood group may also represent a link between cardiovascular risk and cognitive function. A recent large cohort study demonstrated that blood group AB and higher FVIII were associated with increased incidence of cognitive impairment [10].

The relation between the ABO blood group and hemostasis has been predominately attributed to the fact that in non-O individuals the plasma levels of FVIII-von Willebrand Factor (VWF) complexes are approximately 25% higher than in O individuals [9, 11], as it is known that elevated VWF and FVIII levels are associated with venous thromboembolism and coronary heart disease whereas low levels cause a bleeding tendency [4, 12–16].

VWF not only promotes platelet adhesion and aggregation but it is also a key player in the exposure of procoagulant phospholipids on the membrane of activated platelets and is therefore essential in thrombin generation (TG) in platelet rich plasma (PRP) [17]. Also, VWF is the specific carrier of FVIII in plasma and protects it from proteolytic degradation, prolonging its half-life in circulation and efficiently localizing it at the site of vascular injury [18]. FVIII plays a crucial role in the propagation phase of coagulation activation as an essential cofactor in the activation of FX by FIXa and thus is essential for thrombin generation in both PRP and PPP [19]. It therefore is not unlikely that both factors influence the hemostatic-thrombotic system via their influence on the generation of thrombin.

Thrombin generation is the resultant of prothrombin activation and thrombin inactivation. The conversion of prothrombin into thrombin is a complex event that includes the stepwise activation of coagulation factors, regulated by many positive and negative feedback reactions. Thrombin inactivation is a much simpler process that involves mainly antithrombin (AT), α_2_Macroglobulin (α_2_M) and in which fibrin(ogen) plays a modulating role. In contrast to prothrombin conversion [20], thrombin inactivation can be faithfully modelled in a validated computer model [21], and hence, once the concentrations of AT, α_2_M and fibrinogen are known, the course of prothrombin conversion and thrombin inactivation can be obtained from an overall thrombin generation curve.

It has been acknowledged that there is a significant correlation between the amount of thrombin formed and the propensity towards thrombotic or bleeding states [22–31]. High levels of factors II, VII and VIII result in higher thrombin generation and have been found to correlate with the occurrence of venous thrombosis and myocardial infarction. Elevated VWF levels increase TG in PRP and are a risk factor for arterial thrombosis [4, 12–16].

The differences in VWF and FVIII levels between the ABO groups are thought to be caused by ABH antigenic determinants that are present on the N-linked oligosaccharide chains of circulating VWF and FVIII [32–35], which presumably affect the clearance of the proteins [36–39]. While the relationship of ABO blood group to levels of VWF and FVIII is well recognized, other, yet unrecognized clotting proteins, might also contribute to the differences between blood groups by influencing thrombin generation. Besides VWF and FVIII, thrombin inhibitor α_2_Macroglobulin is the only plasma protein known to have N-linked ABH antigens [32]. Intriguingly, it has been shown that indeed α_2_M levels are higher in blood group O compared to blood group A [40]. Special attention to the process of thrombin inactivation is therefore warranted.

The rationale of the work presented here is to search for a correlation between ABO blood group and thrombin generation in PPP and PRP and to investigate whether any difference that might be found is located in the platelets, in the prothrombin activation system or in the thrombin inactivation reactions. The aim of this study is to investigate the effect of ABO blood group on TG, prothrombin conversion, and thrombin inactivation in relation to the relevant clotting factors VWF, FVIII, α_2_M, antithrombin, prothrombin and fibrinogen.

## Materials and Methods

### Study population

The study was approved by the local ethics committee of the King Abdulaziz University, and carried out according to the declaration of Helsinki. The study included 220 healthy, male subjects in Jeddah (Saudi Arabia), after giving their written informed consent, which was documented at the King Abdulaziz University as approved by the local ethics committee. The ABO blood group distribution was as follows: 98 (45%) group O, 76 (35%) group A, 31 (14%) group B and 12 (6%) group AB, which is normal in Saudi Arabia. The age of the subjects was 30 years on average (± 9 years) and their mean body weight was 74 kg (± 11 kg), and no difference was found between blood groups (p = 0.93 and p = 0.85, respectively).

### Sample handling

Blood was collected into vacutainer tubes (0.105 M trisodium citrate; BD Vacutainer System, UK). Platelet rich plasma (PRP) and platelet poor plasma (PPP) were prepared by centrifuging the whole blood at 150 ∙ g or 2900 ∙ g for 10 minutes at room temperature, respectively.

### Thrombin generation

Calibrated Automated Thrombinography (CAT) was performed as previously described in PPP and PRP [41]. All wells contained 80 μl PRP or PPP and 20 μl of tissue factor (TF; 1 or 5 pM) and phospholipids (4 μM). To calibrator wells, 20 μl of calibrator was added instead. Thrombin generation (TG) was initiated by the addition of 20 μl of ZGGR-AMC (417 μM) and CaCl_2_ (16.6 mM). The TG fluorescence data was converted to thrombin generation curves, as described elsewhere [42].

### Coagulation factors determinations

The automated coagulation analyser STA-R (Diagnostica Stago, France) was used to determine plasma coagulation factor levels, according to manufacturer specifications and standard laboratory methods [43]. Plasma VWF concentrations were determined in an immunologic assay. Functional AT levels were measured in a chromogenic assay. Prothrombin and FVIII levels were measured using a clot-based assay, and fibrinogen levels were determined with the Clauss method [44]. Functional α_2_M levels were determined as previously described[21]. The protocol was adapted to be performed on a STA-R analyser (Diagnostica Stago, France) and the intra-assay and inter-assay CVs are 5% and 12%, respectively.

### Calculation of prothrombin conversion and thrombin inactivation

Thrombin decay was predicted by the previously described and validated computational model [21]. This model describes the rate of thrombin removal in time based on the plasma AT, α_2_M and fibrinogen level and the thrombin generation curve. At any moment during the course of the thrombin generation process, the net velocity of thrombin formation (d(T)/dt) is the result of the velocity of prothrombin conversion (-d(P)/dt) and thrombin decay by its inhibitors (d(T-inh)/dt) (Eq 1). Therefore, if TG is measured in a sample and thrombin decay can be predicted, prothrombin conversion can be calculated (Eq 2). The values of k_AT_ and kα_2M_ have been estimated previously [21].

d(T)/dt=-d(P)/dt - d(T-inh)/dt Eq. 1

$$\text{- d}\left(\text{P}\right)/\text{dt}=\text{d}\left(\text{T}\right)/\text{dt}+{\text{k}}_{\text{AT}}\cdot \;{\left[\text{AT}\right]}_{\text{t}}\cdot \;{\left[\text{T}\right]}_{\text{t}}+{\text{k}}_{\alpha 2\text{M}}\cdot \;{\left[\alpha_{2}\text{M}\right]}_{\text{t}}\cdot \;{\left[\text{T}\right]}_{\text{t}}$$ Eq. 2

### Statistics

The Statistical Package for the Social Sciences (SPSS) was used to determine the statistical significance of the results. Normality of the data was tested using the Shapiro-Wilk test, and ANOVA or Kruskal-Wallis analysis was used to detect differences in group means, accordingly.

## Results

### ABO blood group and plasma levels of VWF, FVIII, prothrombin, antithrombin, fibrinogen and α_2_-Macroglobulin

**Fig 1:**
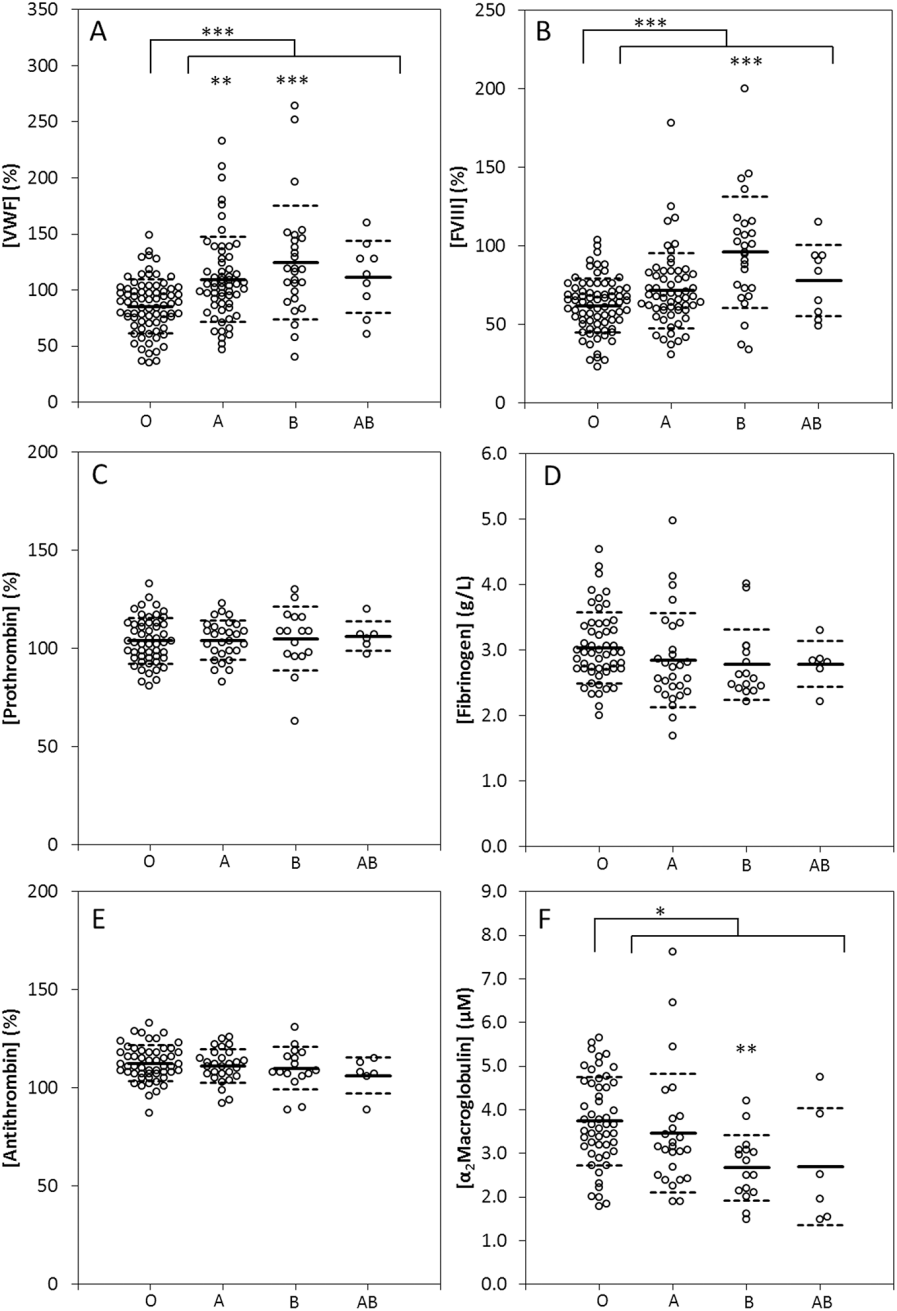
Effect of ABO blood type on the plasma levels of VWF, FVIII, prothrombin, fibrinogen, antithrombin and α_2_M in healthy subjects. VWF (A) and FVIII (B) levels were significantly higher in non O blood groups. Prothrombin (C), fibrinogen (D) and antithrombin (E) levels did not differ between the blood groups, and α_2_M levels (F) were significantly lower in non O blood groups. Data represent individual measurements of donors divided into different ABO groups. * p<0.05, **p<0.01 and ***p<0.001.

It is well documented that the ABO blood group is a major determinant of plasma levels of FVIII and VWF. We confirm that indeed plasma VWF and FVIII levels are increased in non O blood group (Fig 1). VWF levels were approximately 34% higher in the non O blood groups (p<0.001) and FVIII was 27% higher compared to the levels found in blood group O (p<0.001). Plasma levels of prothrombin, fibrinogen, antithrombin and α_2_Macroglobulin were measured, as prothrombin and fibrinogen are known to affect the production of thrombin and its positive feedback, and AT and α_2_M are the major inhibitors of thrombin in plasma. No significant difference in the plasma levels of prothrombin, fibrinogen or AT was seen between the ABO blood groups (Fig 1) and α_2_M levels were higher in blood group O than in the non-O group (122%, p = 0.001).

### Influence of ABO blood group on thrombin generation

**Fig 2:**
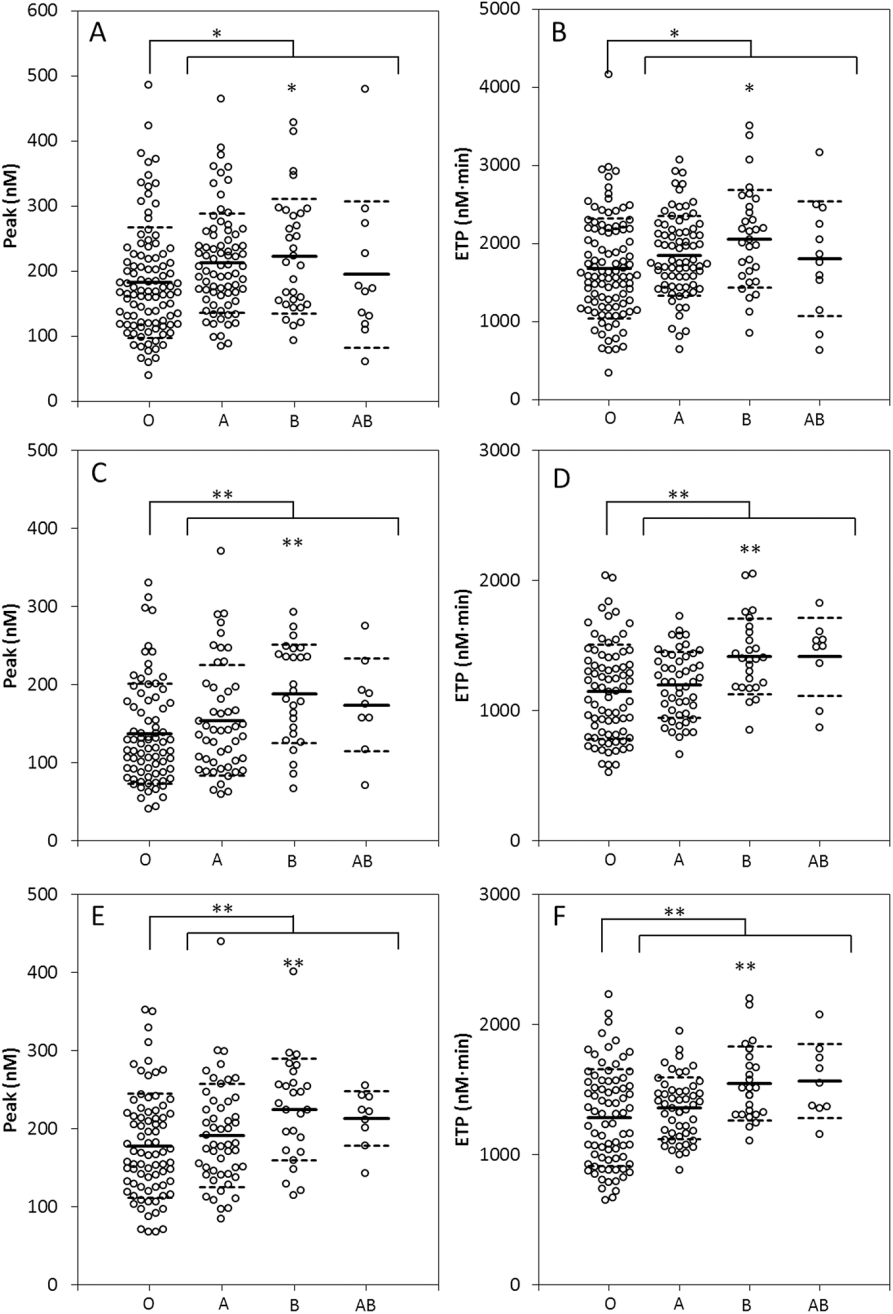
ABO blood type-dependency of the peak height and the ETP in platelet poor and platelet rich plasma. Thrombin generation was measured at 1 pM TF in PRP (A-B) and at 1 pM TF (C-D) or 5 pM TF (E-F) in PPP. The peak height was increased in the non O blood groups in PPP and PRP (A-C-E). The ETP was significantly higher in the non O group, regardless of the TF concentration or the presence of platelets. Data represent individual measurements of donors divided into different ABO groups. * p<0.05 and **p<0.01.

We investigated whether the ABO blood groups had an influence on thrombin generation measured in the presence and absence of platelets. Thrombin generation was triggered with 1 pM TF in PRP and 1 and 5 pM TF in PPP. In Table 1, the mean values of the TG parameters are shown for each blood group. ABO blood group had a significant influence on the peak height and endogenous thrombin potential (ETP), whereas no significant differences were observed for the time-dependent variables i.e. lag time and time to peak (Table 1). The effect of ABO blood group on TG in PPP and PRP was comparable: The ETP was approximately 12% higher in non O blood groups, whereas the peak height was 18% higher. Moreover, the effect of the ABO blood group was comparable at low (1 pM) and high (5 pM) TF concentrations. The mean peak height and ETP (Fig 2) were significantly higher in the non-O group than in the O group.

**Table 1:** Thrombin generation parameters in the ABO blood groups.

	O	A	B	AB	p-value	O/non O (%)
**Platelet rich plasma1 pM TF**	(n = 101)	(n = 76)	(n = 31)	(n = 12)		
Lag time (min)	5.4±1.4	5.2±1.2	5.5±1.2	4.9±0.9	0.462	100
ETP (nM ∙ min)	1682±639	1849±513	2064±628	1810±731	0.018	89
Peak thrombin (nM)	183±85	213±77	224±88	196±112	0.010	86
Time to peak (min)	10.5±2.8	10.0±2.6	10.5±2.6	10.6±2.8	0.574	102
**Platelet poor plasma 1 pM TF**	(n = 79)	(n = 50)	(n = 26)	(n = 9)		
Lag time (min)	4.5±1.0	4.9±1.5	4.5±1.0	4.7±1.1	0.523	93
ETP (nM ∙ min)	1147±361	1199±255	1420±290	1415±301	0.001	90
Peak thrombin (nM)	137±67	154±70	189±63	174±59	0.001	83
Time to peak (min)	9.6±1.6	9.8±2.3	9.1±1.5	10.0±2.1	0.403	99
**Platelet poor plasma 5 pM TF**	(n = 79)	(n = 51)	(n = 26)	(n = 9)		
Lag time (min)	3.0±0.8	3.5±1.7	3.1±0.8	3.1±0.3	0.313	90
ETP (nM ∙ min)	1287±370	1358±237	1549±282	1567±284	0.001	91
Peak thrombin (nM)	178±66	192±66	225±65	213±35	0.006	89
Time to peak (min)	7.7±2.0	8.2±2.7	7.4±1.8	7.8±1.0	0.736	98

Data are presented as mean values ± SD.

### Influence of ABO blood group on prothrombin conversion and thrombin inactivation

**Fig 3:**
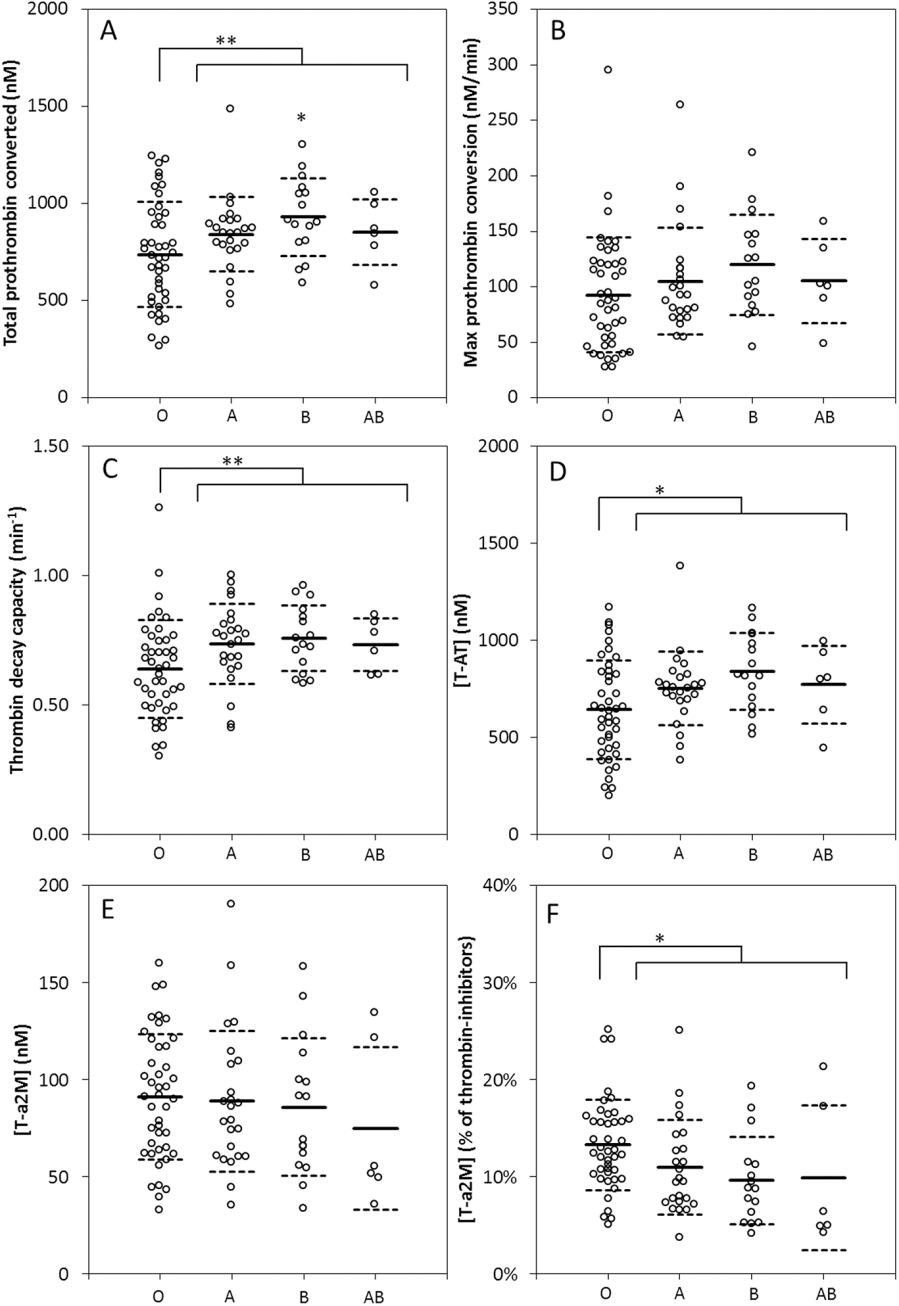
Effect of ABO blood type on prothrombin conversion and thrombin inactivation. The total amount of prothrombin converted was increased in the non-O blood groups (A), but the maximum rate of prothrombin conversion was statistically comparable between the groups (B). The total thrombin decay capacity was increased in the non-O blood group compared to the O group (C). Thrombin-antithrombin (T-AT) formation during TG is significantly increased in the non-O group, whereas the amount of thrombin-α_2_M (T-α_2_M) is comparable (D-E). However, the contribution of α_2_M to the inhibition of thrombin was increased in the O blood group (F). Data represent individual measurements of donors divided into different ABO groups. * p<0.05, **p<0.01 and ***p<0.001.

A change in thrombin generation is caused by a change of its underlying processes: prothrombin conversion and thrombin inactivation. To investigate further how changes in thrombin generation between blood groups arise, we calculated prothrombin conversion and thrombin inactivation curves from thrombin generation data and thrombin inhibitor levels as described under methods. The total amount of prothrombin that was converted during thrombin generation was higher in the non-O blood groups compared to the O blood group (Fig 3A). Although the maximum rate of prothrombin conversion shows a tendency to be higher in the non-O groups, no statistical significance was found (Fig 3B). In addition, the thrombin decay capacity, which is dependent on the antithrombin, α_2_M and fibrinogen levels in plasma, was significantly higher in the non-O blood groups (Fig 3C). The higher prothrombin conversion and thrombin decay capacity of the non-O groups were reflected in increased formation of thrombin-antithrombin ([T-AT]) complexes during thrombin generation (Fig 3D). The total amount of thrombin-α_2_Macroglobulin ([T-α_2_M]) during TG did not differ between the blood groups (Fig 3E), but the relative contribution of α_2_M to the inhibition of thrombin was higher in blood group O (Fig 3F), which corresponds with the higher levels of α_2_M that were detected in the O blood group.

## Discussion

We investigated the thrombin generating capacity and phenotypic correlation in ABO blood groups. We confirm the recent results of Choi et al. [45], who found higher peak values in the non-O blood group than in the O blood group in PPP. We see the same pattern of higher TG profiles in PRP. A likely explanation for the elevated TG in non O groups is that non-O individuals are known to have significantly higher levels of both VWF and FVIII than O individuals [9, 11]. However, the increase in TG cannot be attributed to an increased VWF level, because VWF only affects TG in PRP, but not in PPP, due to its platelet dependent nature [46]. FVIII is a more suitable candidate, as increased FVIII levels in this population correlate with increased thrombin generation peak values (R = .201, p = 0.013). Moreover, B blood carriers showed the highest thrombin generating capacity compared to other ABO blood groups, and this group also had the highest FVIII plasma level. This suggests that increased FVIII levels may explain the high TG in PPP and PRP of the non-O blood groups, as it has been shown that variations of the level of FVIII around the normal range do correlate with thrombin generating capacity (unpublished data). This is corroborated by the fact that the clotting dependent parameters (lag time and time to peak) hardly varied in the ABO groups as it is known that FVIII hardly affects the lag time [47]. The mechanism by which FVIII might cause this effect remains enigmatic, because it is seen also at high TF concentration (5 pM) whereas the classical role of FVIII is in the intrinsic pathway and the Josso loop, that play their role at lower TF concentrations only. *In vitro* experiments in which exogenous FVIII was added to pooled normal plasma show that FVIII increases thrombin generation peak height and ETP in a concentration dependent manner (unpublished data).

The next question is whether the increase of thrombin generation is completely attributable to changes in prothrombin activation–as would be expected when increased FVIII were the sole underlying cause. To address this question, we determined the plasma levels of several other coagulation factors and demonstrated that there is no significant difference in the level of antithrombin, fibrinogen and prothrombin between the ABO blood groups. To our knowledge, no quantitative data exist comparing α_2_M levels in the different blood groups (i.e. A, B, AB and O), except for the study of O’Donnell et al. [40] who compared O and A blood groups. We show here that the concentration of α_2_M in the plasma of all non-O groups is significantly lower than those of the O-group, be it that the concentration in the A carriers was higher than in blood group B and AB. This could be explained by the variation in α_2_M levels between the A genotypes as reported earlier by O’Donnell et al. [40]. We performed genotyping on a subset of the A group individuals, and found that 79% of the subjects had one or two A101 alleles, and that 21% had one or two A102 alleles, which both results in the A1 phenotype [48]. We did not find any statistical differences in coagulation factor levels or thrombin generation parameters between the A101 and A102 genotype individuals. The A201 allele, which would result in an A2 phenotype, was not detected in this cohort of individuals.

The thrombin generation test reflects the balance between thrombin production and inactivation. The production is proportional to plasma levels of prothrombin and influenced by other procoagulant factors, among which FVIII, and fibrinogen [49]. The inactivation is caused by antithrombin, α_2_M and some miscellaneous inhibitors. We used a validated computational approach to obtain the courses of prothrombin conversion (i.e. thrombin production) and thrombin inactivation separately. We found that indeed prothrombin conversion is increased in non-O groups, which may be attributable to increased FVIII levels. Also, the thrombin decay capacity is higher in the non-O group even though α_2_M levels are higher in the O blood group. The higher α_2_M levels in the O blood group did result in a higher relative contribution of α_2_M to thrombin decay. Moreover, computational simulation has pointed out that at physiological antithrombin levels, the α_2_M level would have to increase to 200% of normal if it was the only factor to explain the whole effect on TG as seen in the O blood group [21]. Therefore, we believe that the lower TG in the O group is not only the result of an elevation of α_2_M, but mainly to a lower prothrombin conversion–possibly related to an as yet unexplained effect of FVIII.

This study has several limitations. The most important limitation is that A1 and A2 phenotypes were not determined in the total cohort. However, analysis of a subset of individuals revealed no difference of coagulation factor levels or thrombin generation parameters between the A101 and A102 genotypes, and no individuals with genotype A201 were found. These results were confirmed by a larger study (n = 100) in which no individuals with the A201 genotype were identified (unpublished data). Another limitation is the small number of AB individuals, which is a relatively uncommon ABO blood type. It was previously shown that individuals with blood type AB have the highest VWF and FVIII levels [11, 50]. In this study we did not find a significant difference between AB and B individuals, probably due to the small number of individuals in the AB group.

In conclusion, from our results it appears that there is an association between the thrombin generation and the phenotype of the ABO blood group. Our data suggest that both processes underlying TG (prothrombin conversion and thrombin inactivation) differ with the ABO blood group, which can, at least partly, be due to differences in the levels of FVIII and α_2_M. Intriguingly, this shows that also in a normal population, the capacity to form thrombin is proportional–be it not necessarily linearly proportional—to the capacity to arrest bleeding and the tendency to develop thrombosis. This is confirmed by epidemiological studies that show a relative risk of thrombosis of 0.65 in the O group compared to a risk of 1.15 in the non-O groups, whereas the risk of bleeding in the O group is significantly higher than in non-O individuals [51, 52].

## Supporting Information

S1 File Database containing the thrombin generation parameters, coagulation factor levels and patient characteristics of the subjects in the study. (XLSX)

## Abbreviations

VWF: von Willebrand Factor
TG: Thrombin Generation
α_2_M: α_2_Macroglobulin
PRP: Platelet rich plasma
PPP: Platelet poor plasma
AT: Antithrombin
CAT: Calibrated automated thrombography
ETP: Endogenous thrombin potential
T-AT: Thrombin-antithrombin
T-α_2_M: Thrombin- α_2_M

## References

[1] Medalie JH, Levene C, Papier C, Goldbourt U, Dreyfuss F, Oron D, et al Blood groups, myocardial infarction and angina pectoris among 10,000 adult males. The New England journal of medicine. 1971;285(24):1348–53. Epub 1971/12/09. 10.1056/nejm197112092852404 . 5001056

[2] Gandara E, Kovacs MJ, Kahn SR, Wells PS, Anderson DA, Chagnon I, et al Non-OO blood type influences the risk of recurrent venous thromboembolism. A cohort study. Thrombosis and haemostasis. 2013;110(6):1172–9. Epub 2013/09/27. 10.1160/th13-06-0488 . 24067945

[3] Dentali F, Franchini M. Recurrent venous thromboembolism: a role for ABO blood group? Thrombosis and haemostasis. 2013;110(6):1110–1. Epub 2013/10/19. 10.1160/th13-09-0780 . 24136686

[4] Meade TW, Cooper JA, Stirling Y, Howarth DJ, Ruddock V, Miller GJ. Factor VIII, ABO blood group and the incidence of ischaemic heart disease. British journal of haematology. 1994;88(3):601–7. Epub 1994/11/01. . 7819072 10.1111/j.1365-2141.1994.tb05079.x

[5] Kingsbury KJ. Relation of ABO blood-groups to atherosclerosis. Lancet. 1971;1(7692):199–203. Epub 1971/01/30. . 4099871 10.1016/s0140-6736(71)90945-7

[6] Hall R, Bunch GA, Humphrey CS. The frequencies of ABO blood groups and of secretors of ABH group substances in peripheral arteriosclerosis. Atherosclerosis. 1971;14(2):241–6. Epub 1971/09/01. . 5118615 10.1016/0021-9150(71)90053-0

[7] Horwich L, Evans DA, McConnell RB, Donohoe WT. ABO blood groups in gastric bleeding. Gut. 1966;7(6):680–5. Epub 1966/12/01. 5297336 10.1136/gut.7.6.680 PMC1552641

[8] Evans DA, Horwich L, McConnell RB, Bullen MF. Influence of the ABO blood groups and secretor status on bleeding and on perforation of duodenal ulcer. Gut. 1968;9(3):319–22. Epub 1968/06/01. 5661969 10.1136/gut.9.3.319 PMC1552588

[9] Gill JC, Endres-Brooks J, Bauer PJ, Marks WJ Jr, Montgomery RR. The effect of ABO blood group on the diagnosis of von Willebrand disease. Blood. 1987;69(6):1691–5. . 3495304

[10] Alexander KS, Zakai NA, Gillett S, McClure LA, Wadley V, Unverzagt F, et al ABO blood type, factor VIII, and incident cognitive impairment in the REGARDS cohort. Neurology. 2014;83(14):1271–6. 10.1212/WNL.0000000000000844 25209581 PMC4180487

[11] Jenkins PV, O'Donnell JS. ABO blood group determines plasma von Willebrand factor levels: a biologic function after all? Transfusion. 2006;46(10):1836–44. 10.1111/j.1537-2995.2006.00975.x . 17002642

[12] Koster T, Blann AD, Briet E, Vandenbroucke JP, Rosendaal FR. Role of clotting factor VIII in effect of von Willebrand factor on occurrence of deep-vein thrombosis. Lancet. 1995;345(8943):152–5. Epub 1995/01/21. . 7823669 10.1016/s0140-6736(95)90166-3

[13] Jansson JH, Nilsson TK, Johnson O. von Willebrand factor in plasma: a novel risk factor for recurrent myocardial infarction and death. British heart journal. 1991;66(5):351–5. Epub 1991/11/01. 1747294 10.1136/hrt.66.5.351 PMC1024772

[14] Thompson SG, Kienast J, Pyke SD, Haverkate F, van de Loo JC. Hemostatic factors and the risk of myocardial infarction or sudden death in patients with angina pectoris. European Concerted Action on Thrombosis and Disabilities Angina Pectoris Study Group. The New England journal of medicine. 1995;332(10):635–41. Epub 1995/03/09. 10.1056/nejm199503093321003 . 7845427

[15] Folsom AR, Wu KK, Rosamond WD, Sharrett AR, Chambless LE. Prospective study of hemostatic factors and incidence of coronary heart disease: the Atherosclerosis Risk in Communities (ARIC) Study. Circulation. 1997;96(4):1102–8. Epub 1997/08/19. . 9286936 10.1161/01.cir.96.4.1102

[16] Wahlberg TB, Blomback M, Overmark I. Blood coagulation studies in 45 patients with ischemic cerebrovascular disease and 44 patients with venous thromboembolic disease. Acta medica Scandinavica. 1980;207(5):385–90. Epub 1980/01/01. . 6770584 10.1111/j.0954-6820.1980.tb09743.x

[17] Beguin S, Kumar R, Keularts I, Seligsohn U, Coller BS, Hemker HC. Fibrin-dependent platelet procoagulant activity requires GPIb receptors and von Willebrand factor. Blood. 1999;93(2):564–70. Epub 1999/01/13. . 9885217

[18] Vlot AJ, Koppelman SJ, Bouma BN, Sixma JJ. Factor VIII and von Willebrand factor. Thrombosis and haemostasis. 1998;79(3):456–65. Epub 1998/04/08. . 9531024

[19] Hoffman M, Monroe DM. Coagulation 2006: a modern view of hemostasis. Hematology/oncology clinics of North America. 2007;21(1):1–11. Epub 2007/01/30. 10.1016/j.hoc.2006.11.004 . 17258114

[20] Hemker HC, Kerdelo S, Kremers RM. Is there value in kinetic modeling of thrombin generation? No (unless…). J Thromb Haemost. 2012;10(8):1470–7. Epub 2012/06/02. 10.1111/j.1538-7836.2012.04802.x . 22650179

[21] Kremers RM, Peters TC, Wagenvoord RJ, Hemker HC. The balance of pro- and anticoagulant processes underlying thrombin generation. J Thromb Haemost. 2015;13(3):437–47. Epub 2014/11/26. 10.1111/jth.12798 . 25421744

[22] Al Dieri R, de Laat B, Hemker HC. Thrombin generation: what have we learned? Blood reviews. 2012;26(5):197–203. Epub 2012/07/06. 10.1016/j.blre.2012.06.001 . 22762893

[23] van Veen JJ, Gatt A, Makris M. Thrombin generation testing in routine clinical practice: are we there yet? British journal of haematology. 2008;142(6):889–903. Epub 2008/06/20. 10.1111/j.1365-2141.2008.07267.x . 18564356

[24] Berntorp E, Salvagno GL. Standardization and clinical utility of thrombin-generation assays. Seminars in thrombosis and hemostasis. 2008;34(7):670–82. Epub 2008/12/17. 10.1055/s-0028-1104546 . 19085768

[25] Hemker HC, Al Dieri R, De Smedt E, Beguin S. Thrombin generation, a function test of the haemostatic-thrombotic system. Thromb Haemost. 2006;96(5):553–61. Epub 2006/11/03. doi: 06110553 [pii]. . 17080210

[26] Hemker HC, Beguin S. Phenotyping the clotting system. Thrombosis and haemostasis. 2000;84(5):747–51. Epub 2000/12/29. . 11127849

[27] Baglin T. The measurement and application of thrombin generation. Br J Haematol. 2005;130(5):653–61. 10.1111/j.1365-2141.2005.05612.x . 16115120

[28] Ten Cate H. Thrombin generation in clinical conditions. Thromb Res. 2012;129(3):367–70. Epub 2011/11/15. doi: S0049-3848(11)00557-3 [pii] 10.1016/j.thromres.2011.10.017 . 22079443

[29] Hemker HC, Wielders S, Kessels H, Beguin S. Continuous registration of thrombin generation in plasma, its use for the determination of the thrombin potential. Thromb Haemost. 1993;70(4):617–24. Epub 1993/10/18. . 7509511

[30] Baglin T. Unraveling the thrombophilia paradox: from hypercoagulability to the prothrombotic state. Journal of thrombosis and haemostasis: JTH. 2010;8(2):228–33. Epub 2009/12/01. 10.1111/j.1538-7836.2009.03702.x . 19943876

[31] Adams M. Assessment of thrombin generation: useful or hype? Seminars in thrombosis and hemostasis. 2009;35(1):104–10. Epub 2009/03/25. 10.1055/s-0029-1214153 . 19308898

[32] Matsui T, Fujimura Y, Nishida S, Titani K. Human plasma alpha 2-macroglobulin and von Willebrand factor possess covalently linked ABO(H) blood group antigens in subjects with corresponding ABO phenotype. Blood. 1993;82(2):663–8. Epub 1993/07/15. . 7687165

[33] Matsui T, Titani K, Mizuochi T. Structures of the asparagine-linked oligosaccharide chains of human von Willebrand factor. Occurrence of blood group A, B, and H(O) structures. The Journal of biological chemistry. 1992;267(13):8723–31. Epub 1992/05/05. . 1577715

[34] Sodetz JM, Paulson JC, McKee PA. Carbohydrate composition and identification of blood group A, B, and H oligosaccharide structures on human Factor VIII/von Willebrand factor. The Journal of biological chemistry. 1979;254(21):10754–60. Epub 1979/11/10. . 315409

[35] Hironaka T, Furukawa K, Esmon PC, Fournel MA, Sawada S, Kato M, et al Comparative study of the sugar chains of factor VIII purified from human plasma and from the culture media of recombinant baby hamster kidney cells. The Journal of biological chemistry. 1992;267(12):8012–20. Epub 1992/04/25. . 1569060

[36] Franchini M, Mannucci PM. ABO blood group and thrombotic vascular disease. Thrombosis and haemostasis. 2014;112(6). Epub 2014/09/05. 10.1160/th14-05-0457 . 25187297

[37] Casari C, Lenting PJ, Wohner N, Christophe OD, Denis CV. Clearance of von Willebrand factor. Journal of thrombosis and haemostasis: JTH. 2013;11 Suppl 1:202–11. Epub 2013/07/17. 10.1111/jth.12226 . 23809124

[38] Preston RJ, Rawley O, Gleeson EM, O'Donnell JS. Elucidating the role of carbohydrate determinants in regulating hemostasis: insights and opportunities. Blood. 2013;121(19):3801–10. Epub 2013/02/22. 10.1182/blood-2012-10-415000 . 23426946

[39] Davies JA, Collins PW, Hathaway LS, Bowen DJ. von Willebrand factor: evidence for variable clearance in vivo according to Y/C1584 phenotype and ABO blood group. Journal of thrombosis and haemostasis: JTH. 2008;6(1):97–103. Epub 2007/10/24. 10.1111/j.1538-7836.2007.02809.x . 17949477

[40] O'Donnell J, Boulton FE, Laffan MA. The relationship between plasma concentration of alpha2-macroglobulin and ABO blood group. Thrombosis and haemostasis. 2002;88(1):167–8. Epub 2002/08/03. . 12152664

[41] Hemker HC, Giesen P, Al Dieri R, Regnault V, de Smedt E, Wagenvoord R, et al Calibrated automated thrombin generation measurement in clotting plasma. Pathophysiol Haemost Thromb. 2003;33(1):4–15. Epub 2003/07/11. doi: 71636 71636 [pii]. . 12853707 10.1159/000071636

[42] Hemker HC, Kremers R. Data management in thrombin generation. Thromb Res. 2013;131(1):3–11. Epub 2012/11/20. doi: S0049-3848(12)00794-3 [pii] 10.1016/j.thromres.2012.10.011 . 23158401

[43] Flanders MM, Crist R, Safapour S, Rodgers GM. Evaluation and performance characteristics of the STA-R coagulation analyzer. Clinical chemistry. 2002;48(9):1622–4. Epub 2002/08/27. . 12194955

[44] Clauss A. [Rapid physiological coagulation method in determination of fibrinogen]. Acta Haematol. 1957;17(4):237–46. Epub 1957/04/01. . 13434757 10.1159/000205234

[45] Choi Q, Kim JE, Kim SY, Han KS, Kim HK. Influence of ABO type on global coagulation assay results: effect of coagulation factor VIII. Clin Chem Lab Med. 2014 Epub 2014/12/17. 10.1515/cclm-2014-0909 /j/cclm.ahead-of-print/cclm-2014-0909/cclm-2014-0909.xml [pii]. . 25503670

[46] Hemker HC, Giesen P, AlDieri R, Regnault V, de Smed E, Wagenvoord R, et al The calibrated automated thrombogram (CAT): a universal routine test for hyper- and hypocoagulability. Pathophysiology of haemostasis and thrombosis. 2002;32(5–6):249–53. Epub 2003/09/19. 10.1159/000073575 . 13679651

[47] Dielis AW, Castoldi E, Spronk HM, van Oerle R, Hamulyak K, Ten Cate H, et al Coagulation factors and the protein C system as determinants of thrombin generation in a normal population. J Thromb Haemost. 2008;6(1):125–31. Epub 2007/11/09. doi: JTH2824 [pii] 10.1111/j.1538-7836.2007.02824.x . 17988231

[48] El-Zawahri MM, Luqmani YA. Molecular genotyping and frequencies of A1, A2, B, O1 and O2 alleles of the ABO blood group system in a Kuwaiti population. Int J Hematol. 2008;87(3):303–9. 10.1007/s12185-008-0036-0 . 18247104

[49] Wolberg AS, Monroe DM, Roberts HR, Hoffman M. Elevated prothrombin results in clots with an altered fiber structure: a possible mechanism of the increased thrombotic risk. Blood. 2003;101(8):3008–13. Epub 2002/12/31. 10.1182/blood-2002-08-2527 . 12506014

[50] Preston AE, Barr A. The Plasma Concentration of Factor Viii in the Normal Population. Ii. The Effects of Age, Sex and Blood Group. Br J Haematol. 1964;10:238–45. . 14141622 10.1111/j.1365-2141.1964.tb00698.x

[51] Dentali F, Sironi AP, Ageno W, Bonfanti C, Crestani S, Frattini F, et al Relationship between ABO blood group and hemorrhage: a systematic literature review and meta-analysis. Seminars in thrombosis and hemostasis. 2013;39(1):72–82. 10.1055/s-0032-1329550 . 23299820

[52] Franchini M, Favaloro EJ, Targher G, Lippi G. ABO blood group, hypercoagulability, and cardiovascular and cancer risk. Critical reviews in clinical laboratory sciences. 2012;49(4):137–49. 10.3109/10408363.2012.708647 . 22856614

